# Geographical Variations of Volatile Metabolites in Newhall Navel Orange Based on HS-SPME-GC-MS and Meteorological Factors

**DOI:** 10.3390/foods15132313

**Published:** 2026-06-29

**Authors:** Yiwen Hu, Wen Lu, Mengyu Ma, Jun Wang, Yanyan Ma, Yongqiang Zheng

**Affiliations:** 1 Citrus Research Institute, Southwest University, Chongqing 400712, China; ywhu9050@163.com (Y.H.); luwen991218@yeah.net (W.L.); 17825729469@163.com (M.M.); wj1985@swu.edu.cn (J.W.); 2 National Citrus Engineering Research Center, Chongqing 400712, China

**Keywords:** Newhall navel orange, volatiles, HS-SPME-GC-MS, partial least squares discriminant analysis (PLS-DA), meteorological factors

## Abstract

Newhall navel orange, a major citrus variety in China, shows considerable variation in fruit quality across production regions. To investigate the key factors driving the geographical variation, this study systematically compared the quality of Newhall navel oranges from 13 production areas and analyzed the relationships between volatile metabolites and climate variables. Our results revealed pronounced regional differences in both fruit physicochemical properties and volatile profiles. Total soluble solids, titratable acid content, and peel color parameters (L*, a*, b*) were identified as the core physicochemical indicators most strongly associated with quality variation. Using headspace solid-phase microextraction coupled with gas chromatography–mass spectrometry (HS-SPME-GC-MS), 106 volatile organic compounds (VOCs) were identified, of which 56 were selected as potential differential markers via partial least squares discriminant analysis (PLS-DA). Correlation analysis and partial least squares regression (PLSR) revealed that annual mean wind speed (AMWS), mean diurnal temperature variation at the expansion stage (MDTV-ES), and mean wind speed, total sunshine duration, mean diurnal temperature variation at the degreening stage (MWS-DS, TSD-DS, MDTV-DS) were important meteorological factors related to volatile metabolism. The study clarified the geographical variations in physicochemical characteristics and volatile profiles of Newhall navel oranges, as well as the key climatic factors linked to volatile metabolism, providing a crucial theoretical basis for site-specific cultivation planning, demarcation of high-quality production areas, and targeted quality regulation of citrus varieties.

## 1. Introduction

Navel orange (*Citrus sinensis* Osbeck var. *brasiliensis* Tanaka), a member of the *Citrus* genus in the Rutaceae family, is an economically important fruit crop in China’s citrus industry. The species encompasses a range of varieties, with major cultivated types including Newhall, Fukumoto, Cara Cara, and Lane Late navel oranges [1,2,3]. Among these, Newhall navel orange originated in California as a bud mutation of the Washington navel orange. Valued for its early ripening, high yield, good storability, and strong market appeal, it has become the most widely grown sweet orange variety in China. Since its introduction in 1978, Newhall navel orange has been extensively developed in regions including Jiangxi, Hubei, Chongqing, Sichuan, Guangxi, and Hunan [4,5]. However, with the expansion of cultivation, considerable variation in fruit quality has emerged across production areas due to differences of environmental conditions, such as climate and soil. This variation adversely affects consumer acceptance and hinders the standardization and branding of the industry.

As core regulators of plant growth, development, and quality formation, climatic conditions exert a profound influence on fruit physicochemical properties, such as soluble solid–acid ratio, peel color, and firmness, as well as the composition and accumulation of volatile organic compounds (VOCs) [6,7,8]. Previous studies have showed that the effects of different climatic factors on fruit volatile metabolism can vary substantially. For instance, high temperatures promote the accumulation of volatile alcohols in grapes while reducing phenolic and terpenoid content; low temperatures during the last month before harvest could be associated with higher concentrations of volatiles in blackcurrants [9,10]. The concentrations of terpenoids such as α-pinene, β-pinene, and d-limonene in apples show a significant negative correlation with growing-season precipitation, while higher rainfall promotes the accumulation of aliphatic aldehydes in grapes [11,12]. Relative humidity and wind speed also play roles in regulating volatile synthesis in fruit. Paterson et al. [13] reported that relative humidity was the primary environmental factor affecting the volatile composition of strawberries, and the daily increase in C9 aldehyde volatiles of cucumbers was significantly negatively correlated with average relative humidity [14]. In addition, wind speed and accumulated temperature have been recognized as important environmental drivers of fruit quality [15,16,17]. Although the regulatory role of climatic factors in fruit quality has been confirmed across various horticultural crops, a systematic analysis of the correlations between meteorological factors, particularly stage-specific climatic parameters during key developmental periods, and the volatile concentrations of Newhall navel oranges remains relatively limited. This knowledge gap hampers the mechanistic interpretation of regional quality differences and the delineation of high-quality cultivation zones.

In recent years, headspace solid-phase microextraction coupled with gas chromatography–mass spectrometry (HS-SPME-GC-MS) has been increasingly employed for the detection of plant volatile components [18,19,20,21]. Compared with other headspace techniques, HS-SPME offers several advantages, including low detection limits, good reproducibility, simple operation, high efficiency, and automation. When coupled with GC-MS, this approach substantially enhances the sensitivity of volatile detection [22]. The integration of complex volatile datasets with multivariate statistical methods such as partial least squares discriminant analysis (PLS-DA) enables the rapid screening of key factors, a strategy that has been proven highly effective for geographical origin discrimination, cultivar identification, cultivation mode evaluation, and processing method optimization in horticultural crops [23,24,25,26]. However, current applications of HS-SPME-GC-MS to Newhall navel oranges have been largely confined to volatile analysis of samples obtained from a single production area or under specific cultivation conditions. Systematic investigation into regional differences in volatile profiles across multiple growing regions in China, as well as their correlations with meteorological factors, have not yet been reported.

In this study, mature fruits from 13 major Newhall navel orange production regions in China were selected for quality analysis. Physicochemical indicators including soluble solids content, titratable acid content, and peel color parameters were measured. The HS-SPME-GC-MS technique was applied to qualitatively and quantitatively detect volatile organic compounds in the juice. PLS-DA models were used to screen key physicochemical indicators and crucial VOCs that contributed most to distinguishing the different growing regions. Furthermore, annual meteorological data as well as stage-specific climatic parameters during two key developmental phases, fruit expansion and degreening, were collected for each of the 13 regions. The Pearson correlation analysis and partial least squares regression analysis were employed to examine associations between meteorological variables and the concentrations of juice VOCs. In parallel, a sensory evaluation was conducted to assess consumer preferences for the flavor of Newhall navel orange fruits from different origins. The overarching aims of this study were to clarify geographical differentiation of volatile metabolic profiles in Newhall navel oranges, identify the key meteorological factors that may influence volatile accumulation, and provide a theoretical basis and practical guidance for delineating high-quality production zones, enabling targeted quality improvement and promoting the standardized industrial development of Newhall navel oranges in China.

## 2. Materials and Methods

### 2.1. Fruit Sample Collection

**Figure 1:**
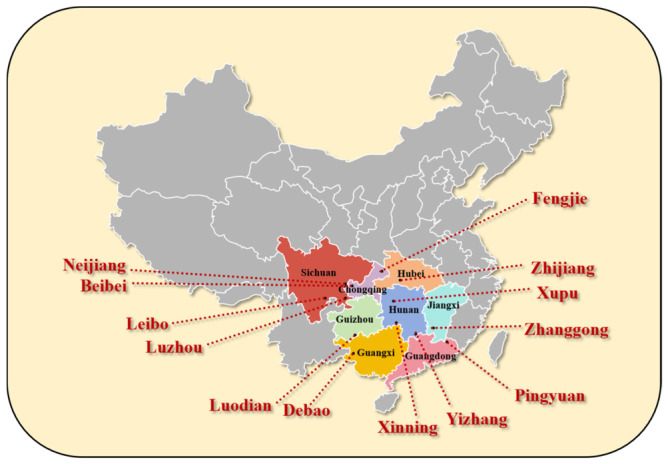
Geographical locations of the 13 sampling regions.

Newhall navel orange fruits were harvested at commercial maturity from 13 major production regions across China. These included Beibei District (BB) and Fengjie County (FJ) in Chongqing; Luzhou City (LZ), Neijiang City (NJ), and Leibo County (LB) in Sichuan Province; Xupu County (XP), Xinning County (XN), and Yizhang County (YZ) in Hunan Province; Zhanggong County (ZG) in Jiangxi Province; Zhijiang City (ZJ) in Hubei Province; Luodian County (LD) in Guizhou Province; Pingyuan County (PY) in Guangdong Province; and Debao County (DB) in the Guangxi Zhuang Autonomous Region (Figure 1). To minimize sampling error, three standardized orchards with consistent management practices (i.e., uniform annual fertilization, irrigation methods, and pest control measures) were selected in each production area. All experimental trees were 12–15 years old and grafted onto trifoliate orange (*Poncirus trifoliata*) rootstocks, with a planting spacing of 3 m × 4 m. Detailed orchard information, including geographic coordinates (latitude/longitude) and harvest date, is provided in Table S1. Within each orchard, six healthy, mature fruit-bearing trees of uniform size and vigor were selected. From each tree, 5–10 fruits were randomly collected from each canopy direction (east, west, south, north, and center) following a “five-point sampling method”. Only fruits with uniform peel color and no visible defects were selected for analysis.

### 2.2. Determination of Fruit Physicochemical Indicators

External quality parameters, including single-fruit weight, vertical and horizontal diameters, peel color indices (L*, a*, b* values), and peel thickness, were measured following the method described by Singh et al. [27]. Juice soluble solids content (SSC) was determined using a digital refractometer (PAL 1; Atago, Tokyo, Japan) [28]. Titratable acid (TA) content and ascorbic acid (vitamin C) content were measured using NaOH titration and the 2,6-dichlorophenolindophenol sodium salt titration method, respectively [7,29].

### 2.3. Sensory Evaluation of Newhall Navel Orange Juice

To evaluate the sensory characteristics and consumer preference for Newhall navel oranges from different regions, a panel of 30 professionally trained assessors (aged 22–45 years; male-to-female ratio of 2:3) performed sensory evaluations of juice samples based on visual observation, olfactory assessment, and taste perception. The sensory evaluation was conducted in accordance with the agricultural standard “Fruit Juice Determination Methods: Sensory Evaluation” (NY 82.2-1988) [30]. For each region, 30 mL of freshly squeezed juice was placed in a transparent plastic cup labeled with a three-digit code (blind-coded, without region names). Assessors scored the samples for aroma intensity (0–100), taste intensity (0–100), color liking (−100 to 100), and overall liking (−100 to 100), where −100 indicated the strongest dislike and 100 indicated the strongest liking [31].

### 2.4. Detection of Juice Volatile Components by HS-SPME-GC-MS

Volatile compounds in the juice were analyzed using gas chromatography–mass spectrometry (8890-7000E GC-MS, Agilent, Santa Clara, CA, USA) combined with headspace solid-phase microextraction (HS-SPME; PAL RSI 120, Agilent, USA). After removing the peel, the fruit pulp was squeezed with a juice extractor. Then, 5 mL of freshly squeezed juice and 1.8 g of NaCl (≥99.5%, psaitong, Beijing, China) were placed in a 20 mL headspace vial, to which 1.5 µL of cyclohexanone internal standard solution (14.25 µg/mL) was added. After mixing, the vial was equilibrated at 40 °C with stirring at 600 rpm for 20 min. An SPME fiber (120 μm DVB/Carbon WR/PDMS, Agilent, USA) was then inserted into the vial to adsorb volatiles for 30 min. The fiber was subsequently transferred automatically by the autosampler (PAL RSI 120, Agilent, USA) to the GC inlet, where desorption was performed at 250 °C for 5 min. Separation of volatile compounds was achieved using an HP 5MS UI capillary column (30 m × 0.25 mm, 0.25 μm, Agilent, USA). High-purity helium (99.999%) served as the carrier gas at a constant flow rate of 1 mL/min. The temperature program was as follows: initial oven temperature 35 °C, held for 5 min; increased to 180 °C at 3 °C/min and held for 2 min; then increased to 250 °C at 5 °C/min and held for 2 min. The split ratio was 20:1. The ion source was electron ionization (EI) operated at 70 eV. The ion source temperature was set at 230 °C and the MSD transfer line at 280 °C. Mass spectra were acquired in full-scan mode (*m*/*z* 35–450). Retention indices (RIs) were calculated by linear interpolation using n alkane standards (C_5_–C_25_). Volatile compounds were tentatively identified by matching both mass spectra and RI values against the NIST20 library. Quantitative analysis was performed using the internal standard method.

### 2.5. Meteorological Data Collection

Climatic parameters were collected for the 13 Newhall navel orange production regions, including annual mean temperature (AMT), annual mean diurnal temperature variation (AMDTV), ≥10 °C annual available accumulated temperature (≥10 °C AAAT), annual mean relative humidity (AMRH), annual total precipitation (ATP), annual total sunshine duration (ATSD), and annual mean wind speed (AMWS). In addition, the following stage-specific parameters were obtained for both the fruit expansion stage (ES) and the degreening stage (DS): mean temperature (MT), mean highest temperature (MHT), mean lowest temperature (MLT), mean diurnal temperature variation (MDTV), ≥10 °C available accumulated temperature (≥10 °C AAT), mean relative humidity (MRH), total precipitation (TP), total sunshine duration (TSD), and mean wind speed (MWS). All meteorological data were sourced from the WheatA agricultural meteorological big data system V1.6.7 (https://wheata.cn/).

### 2.6. Statistical Analysis

One-way analysis of variance (ANOVA) and Duncan’s multiple range test were performed on the selected fruit physicochemical and volatile data using R 4.4.3. PLS-DA was conducted using SIMCA 14.1 (Umetrics AB, Umeå, Sweden). Visualization plots and Pearson correlation analyses were generated using Origin 2021 (OriginLab, Northampton, MA, USA) and the ChiPlot online platform (https://www.chiplot.online). Partial least squares regression (PLSR) analysis was performed using the SPSSPRO online platform (https://www.spsspro.com/).

## 3. Results

### 3.1. Physicochemical Characteristics of Newhall Navel Orange Fruits from 13 Regions

**Figure 2:**
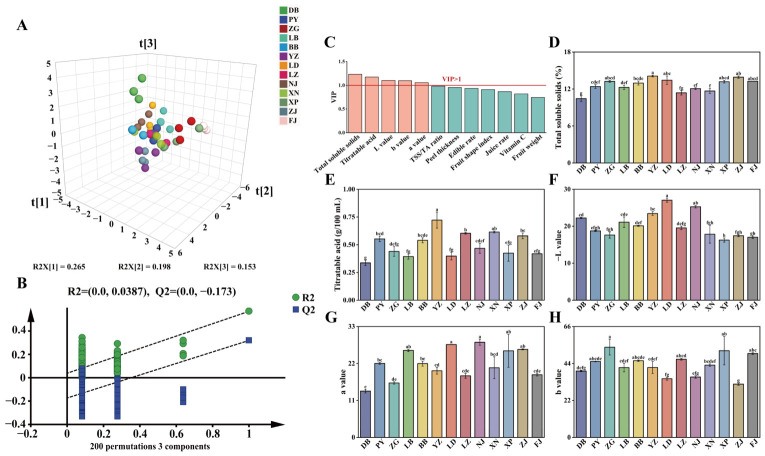
Comparison of physicochemical properties of Newhall navel oranges from 13 production areas. (**A**) Partial least squares discriminant analysis (PLS-DA) score scatter 3D plot based on fruit quality variables. Abbreviations: DB, Debao; PY, Pingyuan; ZG, Zhanggong; LB, Leibo; BB, Beibei; YZ, Yizhang; LD, Luodian; LZ, Luzhou; NJ, Neijiang; XN, Xinning; XP, Xupu; ZJ, Zhijiang; FJ, Fengjie (used throughout). (**B**) Permutation test plot of the PLS-DA model. The two dashed lines represent the regression lines of R2Y and Q2, respectively. (**C**) VIP scores of physicochemical variables in the PLS-DA model. (**D**–**H**) Physicochemical quality attributes, total soluble solids content, titratable acid content, and peel color indices (L*, a*, b*) of fruits from the 13 regions. Lowercase letters indicate significant differences (*p* < 0.05) based on Duncan’s multiple range test.

To comprehensively evaluate the physicochemical characteristics of Newhall navel orange fruits from different growing areas, a PLS-DA model was constructed based on measurements of external quality parameters, including single-fruit weight, fruit shape index, peel color indices (L*, a*, b*), and peel thickness, and internal quality parameters, including soluble solids content, titratable acid content, and ascorbic acid content (Table S3). As shown in Figure 2A, samples from the 13 regions were clearly separated in the 3D score plot, indicating pronounced geographical differences in fruit physicochemical properties. A permutation test (200 iterations) confirmed the robustness of the model. All permuted Q^2^ values (blue) and R^2^ values (green) on the left were lower than the original values on the right, and the Q^2^ intercept was negative, demonstrating no overfitting (Figure 2B). Based on thresholds of variable importance in projection (VIP) score > 1 and *p* < 0.05, five physicochemical properties were identified as key discriminators among regions: soluble solids content, titratable acid content, and peel color L*, a*, and b* values (Figure 2C).

Specifically, the SSC of fruits from the 13 regions ranged from 10.45% to 14.10%. The SSC in samples from YZ, ZJ, LD, ZG, FJ, and XP exceeded 13%, higher than those from other regions. Samples from DB exhibited the lowest SSC (Figure 2D, Table S4). For titratable acidity (TA), YZ showed the highest value (0.72 ± 0.07 g/100 mL) and DB the lowest (0.34 ± 0.02 g/100 mL) among all samples (Figure 2E, Table S4).

Peel color, commonly quantified by the parameters including L*, a*, and b*, is a key quality attribute that warrants close attention due to its substantial impact on citrus fruit appearance and consumer preference. The L* value, representing lightness, varied significantly across regions. As shown in Figure 2F, LD exhibited the lowest L* value (−27.05 ± 0.56), indicating the darkest peel color, whereas XP showed the highest value (−16.26 ± 0.56), indicating the greatest lightness. Notably, no significant difference in L* value was observed among PY, ZG, LZ, XN, ZJ, and FJ (Figure 2F, Table S4). The a* value, which corresponds to the red–green spectrum, is positively correlated with red intensity. Samples from NJ, LD, ZJ, LB, and XP displayed relatively high a* values, suggesting a pronounced red tone in the peel. In contrast, samples from DB, ZG, LZ, and FJ showed lower a* values (Figure 2G, Table S4). High b* values were observed in samples from ZG, XP, FJ, LZ, BB, and PY, implying a deeper yellow tone. Conversely, samples from ZJ, LD, NJ, and DB all had b* values below 40 (Figure 2H, Table S4).

### 3.2. Regional Differences in the Sensory Profile of Newhall Navel Orange Juice

**Figure 3:**
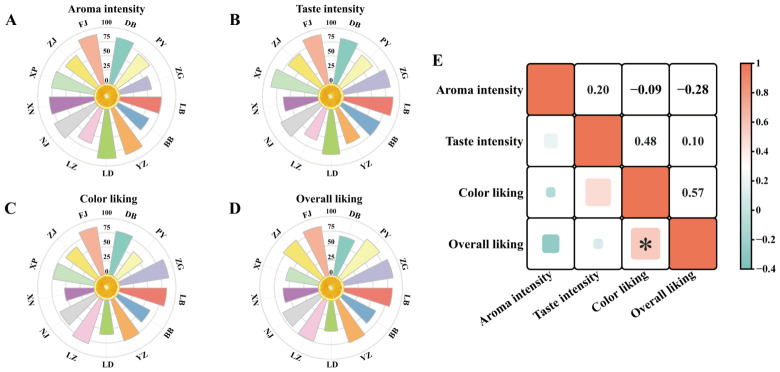
Sensory attribute scores of Newhall navel orange juice from 13 regions. (**A**) Aroma intensity scores. (**B**) Taste intensity scores. (**C**) Color liking scores. (**D**) Overall liking scores. (**E**) Correlation analysis among sensory attributes. An asterisk (*) indicates *p* < 0.05; numbers in boxes are Pearson correlation coefficients.

A trained panel (*n* = 30) conducted a descriptive sensory analysis to evaluate juice samples from Newhall navel oranges of different geographical origins (Table S2). The resulting sensory profiles, illustrated in Figure 3, revealed distinct regional differences. FJ juice received the highest mean score for aroma intensity, whereas BB scored the lowest (Figure 3A). A similar pattern was observed for taste intensity, with FJ juice rated as the most intense, followed in descending order by LB, XP, ZG, DB, and LD (Figure 3B).

In terms of visual preference, juice from ZG, FJ, LB, and LZ received significantly higher ratings for color (Figure 3C). Consumer overall liking, derived from the hedonic scores, indicated a clear preference for ZG. Samples from FJ, LB, and PY were also rated favorably, while juice from LD received the lowest acceptability score (Figure 3D).

Notably, correlation analysis showed a significant positive relationship between color liking and overall liking (Pearson’s r = 0.57, *p* < 0.05; Figure 3E). This finding highlights the substantial influence of juice color, a key visual quality attribute, on consumers’ overall acceptance of Newhall navel orange.

### 3.3. Volatile Profiling of Newhall Navel Orange Juice from Diverse Geographical Origins via HS-SPME-GC-MS

**Figure 4:**
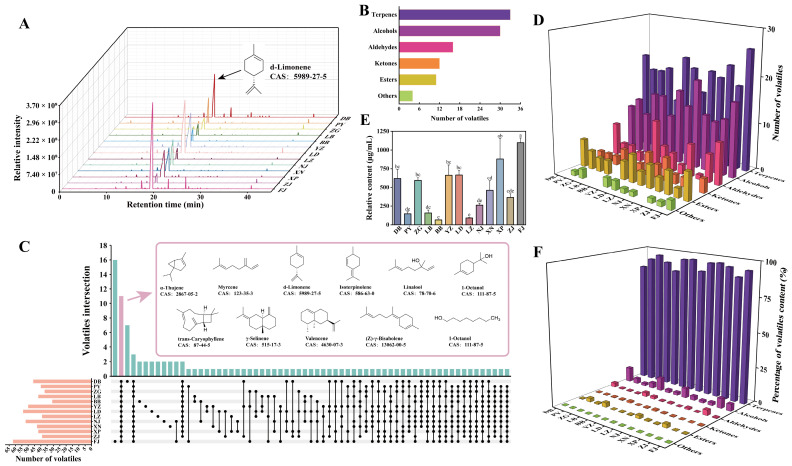
Volatile characteristics of fruit juice from 13 regions. (**A**) Representative total ion chromatogram obtained by headspace solid-phase microextraction coupled with gas chromatography–mass spectrometry (HS-SPME-GC-MS). (**B**) Categories and numbers of volatile compounds detected across all samples. (**C**) UpSet plot showing the distribution of volatile compounds among the 13 regions. The pink arrow indicates the 11 volatile compounds common to all production areas, along with their structural formulas. (**D**) Categories and quantities of volatile compounds in juice from each region. (**E**) Total volatile content in juice from different regions. Lowercase letters indicate significant differences (*p* < 0.05) based on Duncan’s multiple range test. (**F**) Percentage composition of different volatile categories in juice from each region.

The volatile profiles of Newhall navel orange juice from 13 distinct geographical origins were characterized using HS-SPME-GC-MS. Comparison of the total ion chromatograms (TICs) revealed clear regional differences in the composition and abundance of VOCs (Figure 4A).

In total, 106 VOCs were identified and classified into six main categories: terpenes (33 compounds), alcohols (30 compounds), aldehydes (16 compounds), ketones (12 compounds), esters (11 compounds), and others (4 compounds) (Figure 4B, Table S5). Juice from FJ showed the highest VOC diversity (62 compounds), while BB contained the fewest (31 compounds). Eleven volatiles were common to all regional samples, including eight terpenes (α-thujene, myrcene, d-limonene, isoterpinolene, trans-caryophyllene, γ-selinene, valencene, and (Z)-γ-bisabolene) and three alcohols (1-octanol, linalool, and α-terpineol). This common set suggests their potential role as fundamental components of the aroma base of Newhall navel orange juice (Figure 4C).

Terpenes were numerically the dominant chemical class in all regions except BB (Figure 4D). In terms of total volatile content, concentrations ranged from 63.80 μg/L (BB) to 1098.15 μg/L (FJ), representing a 17-fold difference. Juices from LD, YZ, DB, and ZG exhibited intermediate and comparable total volatile concentrations (Figure 4E). Notably, terpenoids dominated the volatile profile across all 13 regions, constituting the largest proportion of identified VOCs. This predominance underscores terpenoids as the principal chemical determinants of the characteristic aroma profile of Newhall navel orange juice (Figure 4F).

### 3.4. Regional Discrimination and Marker Volatiles in Newhall Navel Orange Juice

**Figure 5:**
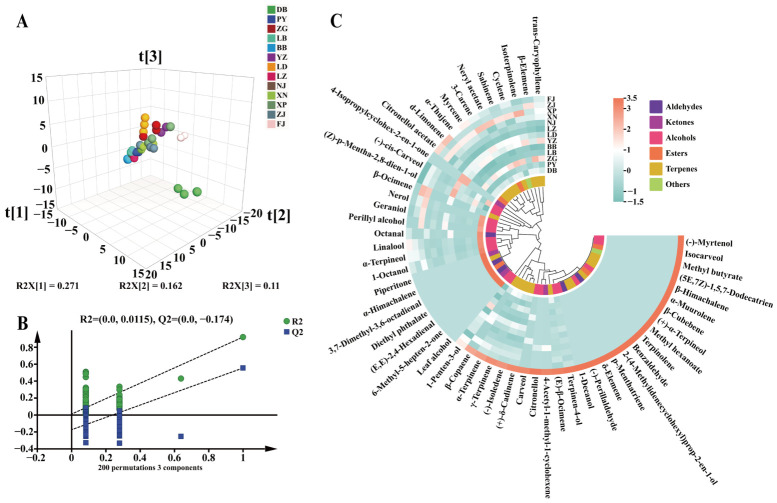
Differential volatile compounds in Newhall navel orange juices from 13 regions. (**A**) PLS-DA score scatter 3D plot based on volatile content. (**B**) Permutation test plot of the PLS-DA model. The two dashed lines represent the regression lines of R2Y and Q2, respectively. (**C**) Heatmap and cluster analysis of the 56 differential volatile compounds (VIP > 1 and *p* < 0.05) in juices from the 13 regions.

A PLS-DA model constructed from volatile compound content data effectively discriminated juice samples by geographical origin. Clear separation was observed in the PLS-DA score plot, particularly for FJ and DB samples, which formed distinct clusters, indicating their unique volatile profiles relative to other origins (Figure 5A). The validity of the model was supported by a permutation test (200 iterations), in which all permuted Q^2^ and R^2^ values remained below the original values, and the corresponding regression lines for both R^2^ and Q^2^ displayed positive slopes, confirming the absence of overfitting (Figure 5B).

Based on the criteria of VIP > 1 and statistical significance of *p* < 0.05, 56 volatile compounds were identified as key markers for regional discrimination. These differential volatiles comprised 22 terpenes, 18 alcohols, 5 aldehydes, 4 ketones, 5 esters, and 2 other compounds. A detailed comparative analysis revealed that 28 volatiles, notably d-limonene, δ-elemene, p-menthatriene, and β-cubebene, occurred at significantly higher concentrations in FJ juice. In contrast, 15 compounds, including 3,7-dimethyl-3,6-octadienal, (E,E)-2,4-hexadienal, and 6-methyl-5-hepten-2-one, were markedly more abundant in DB juice. Notably, 16 components such as isocarveol, (−)-myrtenol, and α-muurolene were detected exclusively in FJ juice, while 7 components including diethyl phthalate, piperitone, and leaf alcohol were unique to DB samples, establishing them as promising region-specific chemical markers.

Furthermore, juices from LD and YZ shared similar accumulation patterns for a set of 10 volatiles, including cyclene, sabinene, neryl acetate, 3-carene, myrcene, α-thujene, d-limonene, citronellol acetate, 4-isopropylcyclohex-2-en-1-one, and β-copaene. XP juice was characterized by a higher relative abundance of specific terpenes, including myrcene, 3-carene, isoterpinolene, and the ester neryl acetate (Figure 5C, Table S5). These region-specific volatile properties not only provide a robust basis for chemical authentication of origin but may also serve as fundamental drivers of the distinctive aroma and flavor profiles associated with Newhall navel oranges from different growing regions.

### 3.5. Analysis of Climatic Characteristics Across 13 Regions in 2024

**Figure 6:**
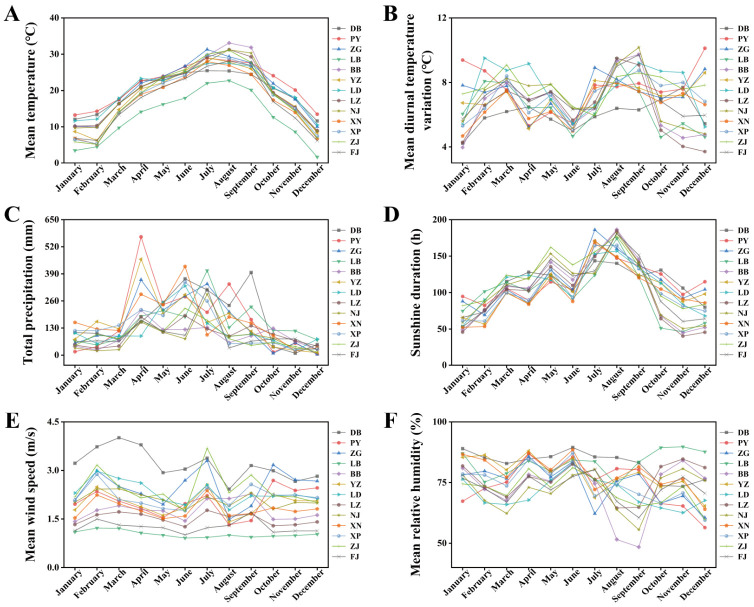
Meteorological conditions in the 13 production regions during 2024. (**A**) Monthly mean temperature of the 13 regions. (**B**) Monthly mean diurnal temperature variation of the 13 regions. (**C**) Monthly total precipitation of the 13 regions. (**D**) Monthly sunshine duration of the 13 regions. (**E**) Monthly mean wind speed of the 13 regions. (**F**) Monthly mean relative humidity of the 13 regions.

Meteorological data were collected from the 13 Newhall navel orange cultivation regions. In addition to annual information, as considered in numerous studies, this study focused on some climatic variables during two critical fruit developmental phases: the fruit expansion stage and the degreening stage (Figure 6, Table 1).

#### 3.5.1. Annual Climatic Characterization

Distinct climatic profiles were observed across the 13 cultivation regions (Figure 6A). The warmest months were consistently July and August, whereas the coolest months were generally February and December, with minimum monthly mean temperatures falling below 10 °C in most regions except DB, PY, and LD. Considerable inter-regional variation was noted in annual mean temperature, which ranged from 12.83 °C (LB) to 21.73 °C (PY). Notably, LB exhibited consistently lower monthly temperatures than all other regions throughout the year (Figure 6A).

A similar trend was observed for annual available accumulated temperature (≥10 °C), which was highest in PY (4348.73 °C·d) and lowest in LB (1034.21 °C·d). In contrast, the annual mean diurnal temperature range showed limited variability (6–8 °C) across all sites (Table 1). Precipitation followed a distinct seasonal pattern, with most rainfall concentrated between April and September (Figure 6C). Annual precipitation in DB and PY was higher than that in other production areas, which generally received less than 2000 mm, while NJ recorded the minimum (1053.16 mm) (Table 1).

Annual cumulative sunshine hours exhibited a clear regional ranking: ZJ (1439.62 h) > PY (1385.48 h) > ZG (1380.84 h) > DB (1321.85 h) > LD (1308.92 h) > FJ (1298.29 h) > NJ (1279.13 h) > YZ (1278.02 h) > XP (1253.29 h) > BB (1245.89 h) > XN (1216.94 h) > LZ (1200.08 h) > LB (1182.69 h). Mean annual wind speed ranged from 0.98 m·s^−1^ to 3.15 m·s^−1^ (Table 1). DB generally experienced higher wind speeds except during July, October, and November, while LB consistently recorded the lowest monthly averages (Figure 6E). Relative humidity was highest in LB (83.58%) and DB (83.28%), with other regions maintaining annual means between 70% and 80% (Table 1).

#### 3.5.2. Climatic Characterization During Critical Fruit Phenological Phases

Pronounced thermal heterogeneity was observed among the cultivation regions during the fruit expansion stage (Table 1). The BB region recorded the highest thermal metrics, with mean, maximum, and minimum temperatures of 31.51 °C, 36.02 °C, and 27.54 °C, respectively. In contrast, the LB region showed the lowest values across all parameters (21.63 °C, 25.82 °C, and 18.15 °C). With the exception of LB, all regions maintained mean temperatures consistently above 20 °C, with minimal fluctuations throughout this period. Compared to annual climatic data, the mean diurnal temperature variation increased notably in all regions during the expansion stage. Regions with higher effective accumulated temperature (≥10 °C) included BB, LZ, NJ, and ZJ, all exceeding 1800 °C·d, while LB accumulated the least heat (1070.31 °C·d). Precipitation during this stage showed substantial regional variability, ranging from 285.35 mm (BB) to 947.43 mm (DB). Despite this variability in water availability, photosynthetically active radiation was relatively uniform, as reflected by a low coefficient of variation (4.99%) for sunshine duration. ZG received the maximum insolation (484.66 h), whereas DB received the minimum (406.37 h). Mean wind speed ranged from 0.9 m·s^−1^ (LB) to 2.96 m·s^−1^ (BB). Monthly mean relative humidity during the fruit expansion stage did not follow a consistent regional trend. Notably, a substantial decrease occurred in the BB region, which may have influenced fruit transpiration rates.

A distinct thermal transition marked the degreening stage, characterized by a general temperature decline across the 13 regions (Table 1). The PY region maintained the most favorable thermal regime, recording the highest mean, maximum, and minimum temperatures. Conversely, LB consistently presented the lowest values across all thermal parameters, establishing a significant thermal gradient important for differential ripening kinetics. Diurnal temperature fluctuation was most pronounced in PY (8.38 °C), while LZ exhibited the most attenuated variation (4.26 °C). Effective accumulated temperature (≥10 °C) differed markedly among regions, with a substantial difference of 742.34 °C·d between the highest (PY) and lowest (LB) values. Precipitation distribution was highly uneven (coefficient of variation: 45.68%). LB received the highest rainfall (309.11 mm), being the only region exceeding 300 mm, whereas both ZG and PY received less than 100 mm. Most regions exhibited a characteristic decline followed by recovery in monthly sunshine hours, with total cumulative insolation highest in PY (339.88 h) and lowest in LZ (149.65 h). Compared to the expansion phase, monthly mean wind speed stabilized during this stage and showed reduced inter-regional variability. Average relative humidity ranged from 63.61% to 89.16%, exhibiting limited regional divergence.

### 3.6. Integrative Analysis of Associations Between Climate and Volatile Metabolome of Newhall Navel Orange Juice

**Figure 7:**
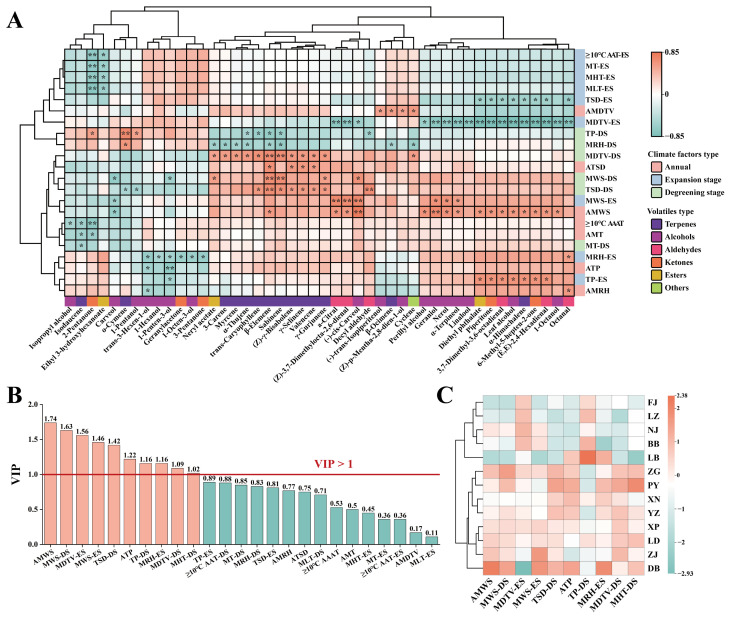
Major meteorological factors influencing volatile metabolism in Newhall navel oranges. (**A**) Correlation analysis and cluster analysis between meteorological factors and volatile content in Newhall navel orange juice. A single asterisk (*) indicates *p* < 0.05; a double asterisk (**) indicates *p* < 0.01. (**B**) VIP values of meteorological factors in the PLSR model. (**C**) Hierarchical clustering heatmap of different Newhall navel orange-producing regions based on meteorological factors. Abbreviations: AMT, Annual mean temperature; AMDTV, Annual mean diurnal temperature variation; ≥10°C AAAT, ≥10°C Annual available accumulated temperature; AMRH, Annual mean relative humidity; AMWS, Annual mean wind speed; ATP, Annual total precipitation; ATSD, Annual total sunshine duration; MT-ES, Mean temperature at the expansion stage; MHT-ES, Mean highest temperature at the expansion stage; MLT-ES, Mean lowest temperature at the expansion stage; MDTV-ES, Mean diurnal temperature variation at the expansion stage; ≥10°C AAT-ES, ≥10°C Available accumulated temperature at the expansion stage; MRH-ES, Mean relative humidity at the expansion stage; TP-ES, Total precipitation at the expansion stage; TSD-ES, Total sunshine duration at the expansion stage; MWS-ES, Mean wind speed at the expansion stage; MT-DS, Mean temperature at the degreening stage; MHT-DS, Mean highest temperature at the degreening stage; MLT-DS, Mean lowest temperature at the degreening stage; MDTV-DS, Mean diurnal temperature variation at the degreening stage; ≥10°C AAT-DS, ≥10°C Available accumulated temperature at the degreening stage; MRH-DS, Mean relative humidity at the degreening stage; TP-DS, Total precipitation at the degreening stage; TSD-DS, Total sunshine duration at the degreening stage; MWS-DS, Mean wind speed at the degreening stage.

To investigate how weather conditions influence the volatile metabolome of Newhall navel orange, a Pearson correlation matrix was constructed linking regional meteorological indices with the quantified volatile profile of mature fruit (Figure 7, Tables S6 and S7). A total of 46 VOCs showed significant (*p* < 0.05) or highly significant (*p* < 0.01) correlations with various climatic parameters. This subset included 14 terpenes, 17 alcohols, 6 aldehydes, 5 ketones, 3 esters, and 1 other compound, reflecting the broad connections between secondary metabolites and environmental signals. Certain meteorological factors exhibited opposite correlation patterns with the accumulation of specific volatiles. For example, the contents of 15 compounds, such as 1-octanol, (E,E)-2,4-hexadienal, 6-methyl-5-hepten-2-one, and α-himachalene, were positively correlated with AMWS but negatively correlated with MDTV-ES. TSD-ES and TP-ES showed significant negative and positive correlations, respectively, with the contents of seven compounds: diethyl phthalate, piperitone, 3,7-dimethyl-3,6-octadienal, leaf alcohol, α-himachalene, 6-methyl-5-hepten-2-one, and (E,E)-2,4-hexadienal.

Thermal parameters during fruit expansion, including ≥10 °C AAT-ES, MT-ES, MHT-ES, and MLT-ES, were consistently negatively associated with 2-pentanone and ethyl 3-hydroxyhexanoate, suggesting that higher heat accumulation may suppress their biosynthesis or accumulation. Interestingly, the concentrations of ten terpenoids (e.g., 3-carene, myrcene, α-thujene) were positively influenced by factors related to evaporation and radiation (MDTV-DS, MWS-DS, TSD-DS, and ATSD) and were negatively influenced by variables affecting ambient moisture (TP-DS and MRH-DS). This pattern closely aligns with known physiological and biochemical responses of terpenoid metabolism to abiotic stress.

In parallel, a PLSR model was constructed to screen key climatic factors contributing most to volatile metabolism of Newhall navel oranges across different regions. The results showed that the major climatic parameters influencing the composition and content of juice volatiles included AMWS (VIP = 1.74), MWS-DS (VIP = 1.63), MDTV-ES (VIP = 1.56), MWS-ES (VIP = 1.46), TSD-DS (VIP = 1.42), ATP (VIP = 1.22), TP-DS (VIP = 1.16), MRH-ES (VIP = 1.16), MDTV-DS (VIP = 1.09) and MHT-DS (VIP = 1.02) (Figure 7B). Utilizing the ten critical meteorological indices, the 13 production regions were categorized into distinct clusters (Figure 7C). ZG, YZ, XP, and LD were characterized by elevated wind speeds, prolonged sunshine duration, and significant diurnal temperature variations. Correspondingly, the fruits from these four regions exhibited similar volatile metabolism profiles.

## 4. Discussion

Newhall navel orange is widely cultivated in China and serves as the principal cultivar for “Gannan Navel Orange”, a product with a Chinese National Geographical Indication designation. In recent years, it has also become a major raw material for the popular beverage “Orange C Americano”, highlighting its substantial economic importance. However, considerable variation in fruit quality across production regions presents challenges for product standardization and industrialization. To address this, the present study systematically evaluated the conventional physicochemical parameters of mature Newhall navel orange fruits from 13 major production areas in China and assessed their sensory attributes through trained panel evaluation. In addition, headspace solid-phase microextraction coupled with gas chromatography–mass spectrometry (HS-SPME-GC–MS) was used to analyze the volatile metabolite profiles of juice from each region. Partial least squares discriminant analysis (PLS-DA) was employed to identify the key physicochemical factors and volatile compounds that contributed most to effectively discriminate fruits of different growing regions. Furthermore, by integrating meteorological data, including both annual indices and stage-specific climatic parameters during two key fruit developmental phases (expansion and degreening), this study examined correlations between climatic variables and volatile content of fruits. The objective was to identify the key climatic factors that significantly influence quality formation in Newhall navel oranges. The findings are intended to provide a valuable scientific basis for guiding high-quality cultivation practices and delineating optimal planting zones.

Fruit quality is shaped not only by genetic factors but also substantially by geographical location and environmental conditions. Multiple studies have confirmed that fruits harvested from different regions often exhibit distinct differences in both external and internal quality traits. For example, ‘Cabernet Franc’ grapes from Rizhao exhibit greater single-fruit weight, larger volume, and higher soluble sugar content, whereas berries from the Taian contain higher total acid content [32]. Similarly, blueberries from Yingkou show higher sugar and lower acid content than those from Weihai [33]. Dried jujubes from western regions such as Xinjiang and eastern Henan are generally considered superior in quality [34]. Additionally, black goji berries from Alxa Left Banner contain higher levels of anti-inflammatory functional compounds, including monogalactosyl diacylglycerol and sulfoquinovosyl diacylglycerol, compared with those from Jinta and Minqin [35]. Consistent with these findings, our study also revealed pronounced geographical variation in the physicochemical properties of Newhall navel oranges across the 13 sampled regions. For instance, fruits from central and southern areas, such as XN, DB, and PY, tended to have greater single-fruit weight. Soluble solids content in juice was generally lower in western production areas (except LD) and in PY in the east, but higher in central regions, a pattern aligning with findings reported by Chen et al. [36]. Apart from BB and LZ, both peel brightness (L* value) and juice vitamin C content were typically reduced in southwestern production regions. Overall, oranges from ZG displayed the most favorable comprehensive quality profile, characterized by high single-fruit weight, near-spherical shape, elevated edible rate and juice yield, high soluble solids and vitamin C content, moderate titratable acid content, a high solids–acid ratio, and rich flavor. These physicochemical results corresponded well with the outcomes of the sensory evaluation.

PLS-DA is a supervised discriminant method that evaluates the contribution of each variable in the model using VIP scores. Higher VIP values indicate that a variable contributes more strongly to distinguishing among sample groups [37]. Based on the physicochemical data of the fruits, a PLS-DA model was established. According to the criteria of VIP > 1 and *p* < 0.05, five key physicochemical parameters were identified: soluble solids content, titratable acid content, and the peel color parameters L*, a*, and b*. These parameters can serve as essential indicators for discriminating Newhall navel oranges of different geographical origins.

VOCs are crucial determinants of fruit flavor quality. Similar to physicochemical properties, the volatile metabolic profiles of fruits also vary across production regions. For instance, total volatile content in winter jujubes from Zhanhua was reported to be higher than in those from Huanghua, Dali, and Yuncheng [38]. Additionally, Qingpi citrus from Hunan exhibited higher diversity and concentration of volatiles than samples from Sichuan, Jiangxi, and Fujian [39]. In Alphonso mangoes, fruits harvested from Deogad contained lower levels of monoterpenes and sesquiterpenes but were richer in lactones and furanones than those from Dapoli [40]. Furthermore, geranyl acetone, 2-pentylfuran, and (E)-β-ocimene have been identified as characteristic VOCs specific to the three main peach-producing regions in Spain: Calanda, Cieza, and Jumilla [41]. Consistent with these findings, our study also revealed distinct regional variations in the composition, diversity, and relative abundance of volatile components in Newhall navel orange juice from the 13 production areas. Notably, juice from FJ exhibited the highest diversity and total concentration of volatile compounds, which may explain its top score in aroma intensity during sensory evaluation. D-limonene, a common compound in Rutaceae plants, possesses antibacterial, insect repellent, insecticidal, and medicinal properties. It is also among the most abundant volatile constituents found in various citrus tissues, including flowers, leaves, peels, and juice sacs [42,43,44,45,46,47].

The combination of volatile compound content with chemometric analysis offers a reliable method for distinguishing the geographical origin of fruits [39,48]. In this study, a PLS-DA model was established based on the volatile content in juice from each production region, and 56 differential volatile compounds were identified that met the criteria of VIP > 1 and *p*-value < 0.05. These differential volatiles consisted mainly of 22 terpenes and 18 alcohols, which are key contributors to the variation in volatile metabolic profiles among production areas and show potential as geographical markers for Newhall navel oranges.

Climatic conditions are critical determinants of fruit metabolite synthesis and accumulation, contributing substantially to the geographical variation in fruit quality observed across different production regions [34]. For example, chestnuts grown in areas with high precipitation, long sunshine duration, and warmer climates show elevated total flavonoid content [49]. Similarly, shorter day lengths and higher relative humidity favor amino acid accumulation in blueberry and blackberry fruits [50]. The metabolite profile of *Amomum villosum* Lour. is closely linked to temperature and precipitation: higher temperatures increase carbohydrate and nucleotide concentrations, while greater precipitation elevates levels of anti-inflammatory active components [51]. In our study, correlation analysis between meteorological data and volatile content from the 13 production areas showed that 46 volatile compounds were significantly correlated with at least one of the 22 climatic variables examined. Among these climatic factors, AMWS and MDTV-ES each displayed significant or highly significant correlations with 17 volatile compounds, indicating their potential influence on the volatile profile.

Specifically, AMWS showed significant positive correlations with the concentrations of 16 volatile compounds, including 1-octanol and (E,E)-2,4-hexadienal, suggesting that higher wind speeds may promote the accumulation of these volatiles, a pattern also observed in *Zanthoxylum bungeanum* [52]. In contrast, greater diurnal temperature variation during the expansion stage appeared to inhibit 17 components, such as α-himachalene and 6-methyl-5-hepten-2-one. Rainfall and diurnal temperature variation are known to affect the volatile composition of ‘Cabernet Sauvignon’ grapes, with aliphatic aldehyde levels significantly correlated with diurnal temperature range [12]. Consistent with this, we found that five aldehydes in Newhall navel oranges, α-citral, (Z)-3,7-dimethylocta-2,6-dienal, 3,7-dimethyl-3,6-octadienal, (E,E)-2,4-hexadienal, and octanal, were negatively associated with MDTV-ES. Notably, both AMWS and MDTV-ES were significantly correlated with the same set of 15 volatile compounds, but exerted opposite relationships with the accumulation of the substances. Moreover, 12 VOCs, including leaf alcohol, 1-octanol, linalool, (E,E)-2,4-hexadienal, and 6-methyl-5-hepten-2-one, were present at higher concentrations in juice from DB than from other regions. This may be explained by DB’s distinct climatic conditions, characterized by high annual mean wind speed and low diurnal temperature variation during the expansion stage.

Additionally, as indicated by PLSR analysis, three climatic parameters of the fruit degreening stage (MWS-DS, TSD-DS, MDTV-DS) were also vital contributors to variations in volatiles of Newhall navel oranges. Volatile metabolism in Newhall navel oranges is probably influenced by diurnal temperature variation, with terpenes generally responding positively to MDTV-DS, while alcohols are predominantly suppressed by MDTV-ES. Reduced daylight duration is often linked to decreased volatile production in plants [53]. In Newhall navel oranges, TSD-DS positively influenced the accumulation of several terpene volatiles. In contrast, TSD-ES generally suppressed the synthesis of terpenes, alcohols, aldehydes, ketones, and esters. This indicates that the same meteorological factor perhaps differentially regulates volatile metabolism depending on the fruit’s developmental stage. Moreover, the concentrations of four specific compounds, γ-gurjunene, γ-selinene, valencene, and (Z)-γ-bisabolene, were higher in orange juice from ZG, XN, and ZJ than in juice from other production regions. This phenomenon probably resulted from the combined influence of several climatic factors, including MDTV-DS, TSD-DS, and MWS-DS.

In contrast to previous studies that have emphasized temperature as a key climatic factor affecting fruit quality formation, our experiment found its regulatory role to be less pronounced [10,54,55]. Instead, wind speed showed a more substantial and significant relationship with both the physicochemical properties and volatile profiles of Newhall navel oranges across the 13 studied production areas. Nevertheless, the specific regulatory effects and mechanisms of wind speed on horticultural crop development, particularly in citrus, remain underexplored in the literature. It should also be noted that the meteorological data used in this study were collected only for the year 2024 and may not fully represent long-term regional climatic patterns. Therefore, the observed correlations between climatic factors and volatile contents require further validation through extended multi-year studies.

Climatic factors play a central role in regulating the metabolism of compounds that determine fruit quality, including sugars, acids, amino acids, and volatile components. This regulation is achieved by modulating gene expression and enzyme activities [43,56]. External stimuli such as light and temperature influence interactions among photosynthesis, carbohydrate synthesis, and pigment metabolism within the fruit. These interactions subsequently affect organic synthesis and oxidoreductase activity, coordinating a series of biological processes during fruit development [57]. Light-regulatory factors maintain mature microRNAs at transcriptional and post-transcriptional levels, which in turn negatively regulate target genes and control terpenoid synthesis [58]. In the carotenoid metabolic pathway, low-temperature stress affects the expression of the structural gene *OfPDS3–2*, thereby influencing the synthesis of β-ionone, a key aroma-contributing compound in *Osmanthus fragrans* [59]. Furthermore, gene modules containing *ERF* and *HSF* are modulated by temperature and light, which subsequently regulate terpenoid levels in grapes [60]. Beyond directly regulating plant substance synthesis and metabolism, rainfall and temperature can also indirectly shape the volatile metabolic characteristics of *Rosa roxburghii* across different regions by influencing pathogen infestation [61]. Currently, the mechanisms by which meteorological factors affect the physicochemical properties and volatile metabolic pathways of Newhall navel orange fruits remain unclear. More comprehensive and in-depth studies are needed to clarify and verify these mechanisms, with particular emphasis on identifying and functionally validating key regulatory genes.

## 5. Conclusions

This study conducted a comprehensive quality assessment of Newhall navel oranges from 13 distinct geographical origins. The results revealed significant regional variations in physicochemical parameters and consumer preference profiles, with samples from ZG and FJ consistently showing superior quality and higher sensory scores. Volatile profiling via HS-SPME-GC–MS demonstrated clear origin-based differences in volatile organic compounds. Juice from FJ contained the most diverse and abundant volatile components. Five physicochemical indices, soluble solids content, titratable acidity, and peel color parameters (L*, a*, b*), along with 56 differential volatile compounds such as leaf alcohol and linalool, were identified as potential markers for distinguishing geographical origin. Combined results of Pearson correlation analysis and PLSR highlighted key climatic factors related to volatile accumulation, including AMWS, MDTV-ES, MWS-DS, TSD-DS, and MDTV-DS. Together, these findings provide a scientific basis for optimizing cultivation practices and guiding regional planting strategies to improve the quality of Newhall navel oranges. Furthermore, this work offers valuable insights for product development and industrial valorization of both fresh fruits and processed derivatives.

## Figures and Tables

**Table 1:** Meteorological factors of the 13 production regions.

Meteorological Factor	DB	PY	ZG	LB	BB	YZ	LD	LZ	NJ	XN	XP	ZJ	FJ	CV (%)
AMT (°C)	19.88	21.73	20.61	12.83	20.3	18.69	20.33	19.97	19.83	17.26	18.2	18.62	17.2	11.47
AMDTV (°C)	6	7.61	7.3	6.44	6.56	7.06	7.6	6.37	7.06	6.59	7.12	7.69	7.12	7.33
≥10°C AAAT (°C·d)	3695.54	4348.73	4081.11	1034.21	3894.25	3526.28	3890.43	3769.72	3734.62	3094.72	3407.89	3570.01	3014.35	22.65
AMRH (%)	83.28	74.95	75.14	83.58	73.35	79.23	71.36	76.62	72.68	79.83	74.96	72.61	73.16	5.11
ATP (mm)	2120.41	2055.28	1826.95	1857.07	1107.79	1852.08	1438.47	1178.71	1053.16	1942.57	1620.12	1132.01	1169.75	24.49
ATSD (h)	1321.85	1385.48	1380.84	1182.69	1245.89	1278.02	1308.92	1200.08	1279.13	1216.94	1253.29	1439.62	1298.29	5.67
AMWS (m/s)	3.15	1.99	2.45	0.98	1.74	1.93	2.28	1.45	2.07	1.76	2.14	2.4	1.14	28.23
MT-ES (°C)	25.1	28.24	29.44	21.63	31.51	27.64	27.19	29.91	29.83	26.46	28.5	29.61	27.24	8.72
MHT-ES (°C)	28.87	32.87	34.17	25.82	36.02	32.12	31.64	34.5	34.46	30.79	32.98	33.77	31.78	7.99
MLT-ES (°C)	22.68	25.03	25.98	18.15	27.54	24.21	23.79	26.05	25.89	23.09	24.89	26.15	23.43	9.25
MDTV-ES (°C)	6.19	7.84	8.19	7.66	8.47	7.91	7.86	8.45	8.57	7.7	8.09	7.62	8.34	7.43
≥10°C AAT-ES (°C·d)	1389.16	1678.05	1788.59	1070.31	1978.59	1622.71	1581.68	1831.7	1824.45	1513.89	1702.18	1803.89	1585.66	13.6
MRH-ES (%)	84.95	79.33	72.69	80.75	59.72	75.27	72.95	69.19	67.46	77.19	71.83	70.76	69.92	8.49
TP-ES (mm)	947.43	717.25	658.49	769.77	285.35	470.63	342.08	357.58	483.06	433.96	371.28	293.18	420.18	39.22
TSD-ES (h)	406.37	454.68	484.66	419.92	483.92	443.73	443.85	475.66	448.6	436.56	462.11	469.61	462.32	4.99
MWS-ES (m/s)	2.96	1.63	2.2	0.9	2.14	1.84	2.15	1.58	2.09	1.86	2.28	2.92	1.26	28.11
MT-DS (°C)	16.65	19.21	16.48	7.62	14.4	14.42	16.45	14.53	14.19	12.65	13.72	13.38	11.84	18.81
MHT-DS (°C)	20.38	23.85	20.61	10.37	16.83	18.53	20.5	16.81	16.89	16.26	17.63	17.5	14.89	17.65
MLT-DS (°C)	13.7	15.47	12.95	5.48	11.94	10.88	12.99	12.55	11.7	9.35	10.09	9.59	8.65	22.18
MDTV-DS (°C)	6.68	8.38	7.66	4.89	4.89	7.65	7.51	4.26	5.19	6.9	7.54	7.92	6.24	19.9
≥10°C AAT-DS (°C d)	618.51	849.02	634.36	106.68	450.14	478.38	612.85	460.19	434.58	366.01	441.27	421.27	311.5	36.2
MRH-DS (%)	75.12	63.61	66.24	89.16	80.03	72.33	65.77	82.75	78.29	72.72	66.71	67.7	73.85	9.93
TP-DS (mm)	108.39	73.39	67.13	309.11	223.93	70.75	169.17	212.55	142.19	148.71	113.16	109.96	177.81	45.68
TSD-DS (h)	317.71	339.88	314.12	155.32	164.03	300.62	264.82	149.65	174.12	282.22	254.87	256.33	213.18	26.26
MWS-DS (m/s)	2.78	2.5	2.84	0.97	1.51	2.1	2.16	1.29	1.91	1.75	2.17	2.09	1.03	30.1

Abbreviations: AMT, Annual mean temperature; AMDTV, Annual mean diurnal temperature variation; ≥10°C AAAT, ≥10°C Annual available accumulated temperature; AMRH, Annual mean relative humidity; AMWS, Annual mean wind speed; ATP, Annual total precipitation; ATSD, Annual total sunshine duration; MT-ES, Mean temperature at the expansion stage; MHT-ES, Mean highest temperature at the expansion stage; MLT-ES, Mean lowest temperature at the expansion stage; MDTV-ES, Mean diurnal temperature variation at the expansion stage; ≥10°C AAT-ES, ≥10°C Available accumulated temperature at the expansion stage; MRH-ES, Mean relative humidity at the expansion stage; TP-ES, Total precipitation at the expansion stage; TSD-ES, Total sunshine duration at the expansion stage; MWS-ES, Mean wind speed at the expansion stage; MT-DS, Mean temperature at the degreening stage; MHT-DS, Mean highest temperature at the degreening stage; MLT-DS, Mean lowest temperature at the degreening stage; MDTV-DS, Mean diurnal temperature variation at the degreening stage; ≥10°C AAT-DS, ≥10°C Available accumulated temperature at the degreening stage; MRH-DS, Mean relative humidity at the degreening stage; TP-DS, Total precipitation at the degreening stage; TSD-DS, Total sunshine duration at the degreening stage; MWS-DS, Mean wind speed at the degreening stage.

## Data Availability

The original contributions presented in the study are included in the article/Supplementary Materials. Further inquiries can be directed to the corresponding authors.

## Supplementary Materials

The following supporting information can be downloaded at: https://www.mdpi.com/article/10.3390/foods15132313/s1, Table S1: Location information and sampling time for the 13 production regions; Table S2: Sensory attribute scores of Newhall navel orange juice from different regions; Table S3: Physical and chemical properties of Newhall navel oranges from different regions; Table S4: Physicochemical indices (VIP > 1) of Newhall navel oranges from different regions; Table S5: Concentration of volatile compounds in Newhall navel orange juice from different regions; Table S6: Correlation coefficient value between meteorological parameters and volatile compounds content; Table S7: Significance *p*-value of correlation between meteorological parameters and volatile compound content.
